# The Public Health Family Impact Checklist: A Tool to Help Practitioners *Think Family*

**DOI:** 10.3389/fpubh.2019.00331

**Published:** 2019-11-12

**Authors:** AliceAnn Crandall, Lynneth Kirsten B. Novilla, Carl L. Hanson, Michael D. Barnes, M. Lelinneth B. Novilla

**Affiliations:** Department of Public Health, Brigham Young University, Provo, UT, United States

**Keywords:** family, practitioner, public health, tool, family engagement, family diversity, family responsibilities, family stability

## Abstract

Families are an important cornerstone of individual and community health across the lifecourse. Not only do families play a role in the development of health, but the family's health is likewise influenced by individual health behaviors and outcomes. Therefore, to improve population health, public health programs must support families. Limited training in family science, as well as lack of instruments to help “think family,” often result in Public Health practitioners feeling ill-equipped to develop programming that supports, targets, and/or involves a diverse range of families. Tools to help public health practitioners *think family* are limited. The Family Impact Checklist is one tool that may help improve the degree to which policies support families. The purpose of this study was to adapt the Family Impact Checklist specifically for use in public health programming efforts. Through a two-round Delphi approach comprised of 17 public health professionals, the Public Health Family Impact Checklist was developed. The adapted Checklist includes 14 items across four *think family* principles: family engagement, family responsibility, family stability and family diversity. We propose that this tool will help practitioners develop high impact, family-friendly programs that ultimately lead to improved individual and community health.

## Introduction

Public health practice is the application of knowledge, skills, and competencies necessary to perform essential public health services (1). These services broadly include assessment, policy development and assurance. Practitioners serving in governmental organizations such as local and state health departments often carry out these services. A recent call to action for public health practitioners acknowledged the importance of multi-sector collaboration in addressing social, environmental, and economic conditions that effect health but stopped short of any discussion on the role of the family (2).

### Why Families Matter to Public Health Practice

Families are the cornerstone of population health (3, 4), and the family atmosphere is important to understanding public health issues (4). The family's role in generating health, promoting healthy choices, and encouraging behavior change, makes the family a vital focus of policy support and public health interventions. Ultimately, what happens in the home, good or bad, resonates in communities and ultimately affects the nation's health (5). Likewise, issues that affect populations affect the family environment. For instance, teen pregnancy, opioid misuse, alcohol and drug dependency, domestic abuse, violence, psychopathology, suicide, and chronic diseases—all bear a family component either due to family influences on the behavior/outcome or through the impact of the health behavior on the family (6–13).

Families produce health (5) not only as a factor of individual biology and genetics but through the environment and lifestyle that families often share within a household. This environment includes dynamics created by family relationships, interactions, beliefs, values, routines, and practices that lead to repeated patterns of behavior that can persist through one's life (14). Families also provide nurturing and care and serve as gatekeepers to healthcare (15). When and where to seek care, how much care to obtain, and the choice to be treated are family decisions based on family values, beliefs, and practices (16). In a testimony to the Senate, Bronfenbrenner (17) explained that “the family is the most powerful, the most humane, and by far, the most economical system known for building competence and character.”

If families are the primary producers of health across the life course then in order to realize impacts relative to prevention, treatment or rehabilitation programming practitioners must strategically consider the family (3, 17, 18). Indeed, *thinking family* is an important learned skill where practitioners reflect on the connection between individuals and families (14). For public health, *thinking family* ensures that programming strengthens, not weakens, the role of parents and other family caregivers. Oftentimes this connection is more easily realized when working with children. For example, in childhood obesity prevention efforts, practitioners must account for the important role of parents when it comes to behavior change around food choices and physical activity at home. However, the important role of family members may be less evident, although no less important, when working with older adults who also require care to address chronic conditions or mental health issues. Despite this important connection for both children and older adults, many public health practitioners continue to be one-dimensional in their implementation of programming by focusing on behavior change at the individual level (19). Assessment tools are one solution to helping practitioners *think family* or pay greater attention to how public health programming is impacting family engagement, responsibility, stability, and diversity.

### Tools to Help Practitioners Involve Families in Public Health Programming

While involving the entire family is critically important for public health success (5), few resources exist to help practitioners *think family* when program planning for children or older adults in public health. Lessons can be learned from mental health services which reveal several promising approaches (20) including but not limited to implementing *think family* practitioner seminars and trainings and using *think family* (e.g., Common Assessment Framework) assessments and processes (e.g., Team Around the Child) in practice (21, 22). One such assessment is the Family-Focused Mental Health Practice Questionnaire, a tool to help providers examine organizational factors and clinician knowledge and skills for working with families (23). Another mental health resource is the “Family focused practice: Actors and enactments” model, which outlines guidelines for practice and policy on how practitioners, consumers, and family members can work together to influence better mental health patient care (24).

Although these tools used in mental health care are informative, their focal point is typically programming efforts for individual patients in clinical settings. Currently, *think family* tools are limited for population-based programming efforts led by health departments, community-based agencies, and other multi-sectoral partners. One promising tool that has relevance for population-focused work is the Family Impact Checklist (25), which was created to guide conversations regarding the degree to which policies support or hinder families (26, 27). The Family Impact Checklist is based on five guiding principles (family responsibility, family stability, family relationships, family diversity, and family engagement) developed by the Coalition of Family Health Organizations for use in “applying the family impact lens in policy and programs and also to practice” (26). The guiding principles provide a framework through which practitioners can carefully consider the impact of policy and practice on the whole family context. Administration of a family impact analysis using the checklist involves bringing together a team of experts who review how well the policy or program meets each guiding principle by examining a series of checklist questions. The results can lead to important discussions and changes to the policy or program to make it more supportive of families (25). To our knowledge, the efficacy of the Family Impact Checklist tool has not been tested, although its theoretical foundation is well-established (27) and family impact analyses are mandatory requirements for legislation introduced in Hong Kong and other locations (28).

### Aims

We developed the Family Impact Checklist and methodology for use across a variety of programs and policies. Although children and/or adult focused public health professionals can use it as they strive to *think family* for their programming, several of the items are less applicable to public health professionals whose focus is on assessment, implementation, and evaluation. Further, there are important components for public health programming that are missing from this checklist. A more specialized family impact checklist for public health would therefore be useful for practitioners engaged in developing and reviewing population health programs to assist public health practitioners to *think family* (5)—whether focused on children or older adults. The purpose of the current study was to use a Delphi process to revise the Family Impact Checklist for public health programming.

## Methods

### Delphi Process for Revising Checklist

We developed a family impact checklist for public health professionals by utilizing a two-round Delphi approach (29, 30) with a small panel of experts (31). The process included: Stage 1: Adaption and Piloting of the Checklist, Stage 2: Delphi Round 1, and Stage 3: Delphi Round 2. The university Institutional Review Board (IRB) deemed that the study did not require ethics approval.

A group of researchers at the RAND Corporation in Santa Monica, California first introduced the Delphi Method in the 1940s to examine whether the scientific use of expert group opinion was superior over individual decision-making in inexact sciences (32). This methodology involves a repetitive process, in which experts are consulted two or more times on the same questions. Experts typically can view the responses of other participants so that in rounds 2 and onward they can reevaluate their initial responses. In an effort to reduce irrelevant information, feedback is controlled. Consensus is not required, but the objective is to achieve a reliable group opinion (32). Thus, an inherent component of Delphi studies is the opportunity for consistency or reliability to be observable between stages, similar to the principle of saturation as done in other forms of qualitative research. Thoughtful expert participant opinion leads to face and content validity.

#### Stage 1: Adaptation and Piloting of the Checklist

An initial version of the Public Health Family Impact Checklist was created by adapting the original version of the Family Impact Checklist developed by Bogenschneider and the Family Impact Institute (26, 27). The authors created the adaptation. The authors are part of the Families and Public Health Collaborative, a consortium focused on incorporating the family as a pathway of public health in practice, research, and teaching. Each member of the team has training and experience working in public health and conducting public health research. To create the initial version, we carefully reviewed each item on the original checklist for relevance to public health practice. We also considered the objectives of public health (e.g., assessment, implementation, and evaluation through a prevention and population perspective) and considered pertinent items to add. This initial version of the survey was then piloted among a group of seven public health promotion representatives from a local county health department. Following input given by these representatives, we made further revisions to the Checklist.

#### Stage 2: Delphi Round 1

Using the revised Checklist from the pilot, we created an online Qualtrics survey that included the following: (1) the revised Checklist in its entirety, including the public health definition of each family impact principle, the checklist question, and the checklist response options; (2) a mechanism allowing participants to comment on the content and utility of each checklist component. The multiple-choice response options were *no change, I have suggested edits, this question should not be included in the checklist/I have some concerns with this question*. We also provided a textbox for further comments. (3) participants were able to suggest additional items for the Checklist.

A group of 27 practitioner experts from the board of directors of the Utah Society of Public Health Educators (USOPHE), county and state public health agencies, non-profit organizations with a population health focus, and healthcare agencies that provided population-based services to communities were recruited via E-mail. Agencies and representatives were selected based on their expertise in health promotion programming and to provide a wide variety of perspectives across different public health organizations and types of organizations (e.g., public vs. private). We identified participants based on the authors' prior experience working with them, and some were referrals from those who we initially invited. We selected this group of experts as they represented the primary target population for the tool. All participants were experts in public health practice and implementation of interventions. However, only a few had expertise in family science. Each participant was offered a $10 Amazon e-gift card incentive to participate. Round 1 was completed in October 2019. Participants were given 2 weeks to complete the survey. Of the 27 invited experts, 17 responded and 15 completed the survey. The respondents included agency and program directors, program coordinators/managers, health educators, and program specialists. Based on the comments from Delphi Round 1, the Checklist was again revised to address participant comments and edits.

#### Stage 3: Delphi Round 2

In Delphi Round 2, the revised Checklist was posted on a Google Doc in early December 2019. In addition to the revised Checklist, the Google Doc contained a table of anonymous comments and responses from Round 1 for all participants to view. Over the course of 11 days, participants who had respondent to all or part of round 1 were allowed to make edits to the revised Checklist using track changes and could also make comments. Further, participants could respond to comments and edits made by other participants. We included prompts to aid participants in reviewing the changes and making edits and comments.

As an incentive for this round, we offered participants a $10 Amazon e-gift card and a drawing for a $20 Amazon e-gift card. Of the 17 invited to make further revisions and comments, 8 responded. Following Round 2, the authors made final edits to the Checklist. In the event of non-consensus on wording changes, the authors made the final decision.

## Results

### Stage 1—Initial Development

The initial Checklist included four principles (family engagement, family responsibility, family stability, and family diversity) and 14 items. Each item included an example application of the item and the response options “Strong,” “Somewhat,” “Very Little,” and “N/A.” Example items include “To what degree are/were families involved as key stakeholders in the development and planning of the program? E.g., Through focus groups, interviews, community meetings, families as volunteers, etc.” and “How well-does the program provide services that are available and accessible (within reason) to a diversity of families? E.g., Culturally sensitive, available to families with special needs, geographically reachable, available to working parents outside of normal working hours, etc.”

### Stage 2—Delphi Round 1

Results from Delphi Round 1 led to revisions in the Checklist introduction, instructions, definitions of each principle, and response options (“Very Well,” “Somewhat Well,” “Not At All,” and “N/A”). For each item, between 14 and 60% of participants made suggested revisions. However, participants were overwhelmingly in favor of the items included: only 1 participant on one item recommended omitting the item. Thus, revisions were made on all items, but all items were retained. After revisions, we moved one item from family engagement to family responsibility.

### Stage 2—Delphi Round 2: Family Impact Checklist for Public Health

In Delphi Round 2, participants suggested further edits to the introduction, the definition used for each principle, and changes to 10 of the 14 items. However, we did not reach full consensus on all changes. The authors arbitrated the non-consensus. Ultimately, we made changes to the Introduction, family responsibility and family diversity definitions, and to six of the items.

The final checklist, called the “Public Health Family Impact Checklist” (hereafter referred to as Checklist), includes four principles and 14 items across the four principles (see Supplement 1). The four principles include family engagement, family responsibility, family stability, and family diversity (see Table 1). Each principle includes a “think family” definition that describes the purview of the principle.

**Table 1:** Description of public health family impact principles and definitions.

**Family impact principle**	**Definition**	**Number of items**
Family engagement	Public health practitioners who “think family” ensure that families are actively involved in all programming phases	5
Family responsibility	Public health practitioners who “think family” plan and deliver programs that support and empower family members to perform their responsibilities. The program also supports the family's choices in performing these responsibilities. Examples of such functions may include family formation, partner relationships, economic and financial support, childrearing, and caregiving	3
Family stability	Public health practitioners who “think family” strive to plan and deliver programs that encourage stability within the family and recognize the importance of family relationships to individual and family health	4
Family diversity	Public health practitioners who “think family” understand that programs can have varied effects on families from different cultures and ethnic backgrounds. Through the program, practitioners acknowledge and respect the diversity of families and do not discriminate against or penalize families based on economic situation, educational attainment, family structure, geographic locale, disability, religious affiliation, or gender and sexual minority status of individual family members	2

The Checklist differs from the original Family Impact Checklist in a few ways. The original Family Impact Checklist (27) includes five principles: the four principles retained here and a principle for family relationships. In the Checklist, items relating to family relationships were deemed important but were embedded within the other four principles (e.g., “How well-does the program recognize that major changes in family relationships and functionality can impact health, may extend over time, and require major support and attention” was included under family stability). Further, the original checklist includes 33 items. Because the original checklist is meant for a wider audience across multiple disciplines, more questions were needed. To facilitate busy public health practitioners using the Checklist, it was important to limit the number of items to fit on two pages. Thus, only items that were most pertinent to public health practice were included. Several of the items are similar to the original checklist wording, but have a specific public health slant. Other items are original to the Checklist (e.g., “To what degree are/were families involved in the development and planning of the program?”). The response options for each item were also changed from the original. Instead of *Strong, Adequate*, and *Limited*, the Delphi team opted for *Very Well, Somewhat Well*, and *Not At All* (with Not Applicable, or *N/A*, continuing to be an option). These new response options were felt to better match the question format of the Checklist.

The Checklist is meant to be used by public health practitioners who may have limited training on how to involve and support families in public health programming. Since the intention was for practitioners to be able to consider the family impact of their planned or existing program internally (e.g., without bringing in an outside family science expert), two things were added to the Checklist that were not present in the original. First, examples were added for all items to ensure clarity on what was being asked and to help practitioners see the relevance of the question to their work. Second, a series of background questions were added before the “checklist” questions as an impetus to help practitioners consider how families may affect the health issue(s) that their programming addresses and to consider interdisciplinary approaches that they may not have been previously aware of. For example, one of these background questions is “Based on current research or your experience, what family behaviors and dynamics affect the health issue?”

### How Practitioners Can Use the Tool

Public health professionals working for government agencies (e.g., a health department), community non-profits, and hybrid medical and public health organizations with a population focus are the primary intended users of the Checklist. Practitioners working on programs that are in the *planning* stages should use the Checklist to help ensure that the program has a positive family impact and to make necessary adjustments where the program plans may fall short. Additionally, practitioners for existing programs can use the Checklist to examine the degree to which the program *thinks family* thus informing changes to the program to ensure a positive family impact. Ideally, due to the family influence on lifelong health and across health issues, practitioners will examine the family impact of all of their programs.

Relevant team members, partners, and other stakeholders on the project should complete the Checklist. It can be completed individually or together. If done individually, all of the relevant parties should come together to discuss the results, including any discrepancies on responses to particular items and the meaning behind their responses. Not all items will be relevant for every program, and a *N/A* response option is available for such cases. However, the *N/A* option should be used judiciously. For example, budget constraints do not automatically make an item not applicable. Ideally, the results will prompt important discussion that may lead to changes in program operations in an effort to be supportive of families. Practitioners should try to ensure programs generally meets all four family impact principles.

Some practitioners may choose to consult with a family science expert as they do their family impact assessment. Although this may yield added insights, it is not essential. The Checklist was created for use by all public health practitioners, regardless of their level of training in family science.

Although the primary audience for the Checklist is public health providers practicing in the community, the tool can also be useful in other settings. For example, the Checklist may be a valuable teaching tool for appropriate family impact and involvement for family practitioners, other clinical providers, and students in schools and programs of public health. Additionally, it can be used in teaching implementation science methods across multiple fields.

## Next Steps to Help Public Health Practitioners *Think Family*

The Checklist helps practitioners *think family* beyond simply assessing the public health programs implemented or offered in each agency or organization site. Practitioners need to also *think family* in the way programs are delivered and evaluated (33). Public health practitioners in the UK (DHSSPS) over the past few years have institutionalized four questions to *think family* during implementation of their programs and services to individuals and families. These questions are contained in the 2011 online guidebook, *Think child, think parent, think family: A guide to parental mental health and child welfare* (21). The guide identifies practices to improve service planning and implementation for improved family impact. Combining the findings from the Checklist, practitioners in the US may learn from these researchers' four questions how to plan and evaluate more purposeful interventions and produce better family health measure: First, “to what degree do I as a practitioner think beyond the issues presented to me by the participant(s), client(s), or patient(s)?” Your primary population may well-include parents or caregivers with dependents, which could positively (or negatively) affect their health. Many individuals respond better when supported by family members. Practitioners can learn to see a more effective view of health outcomes for individuals and populations when they *think family* at every interaction.

Second, practitioners could ask “to what degree am I as a practitioner aware that other family members may help me with information to support the participant, client or patient care, service or treatment?” (21). When reaching one family member, practitioners may plan to provide the family, including children, as much information as possible. That will often mean that various forms of tailored material may need to be made available for these purposes. Practitioners may need to ask as much as appropriate about the family (or the families in a community) so they know what the family understands and how they feel about the situation at hand.

Third, practitioners could ask “to what degree do I as a practitioner consider the well-being and safety of other family members, including children and others who may be dependents?”(21). In some ways, it is easier to simply focus on individual clients, participants or patients and respond uniquely to them. However, the Checklist is built around the assumption that the assets and challenges of a family need to be acknowledged. Practitioners should be aware of, trained for and prepared to intervene when concerns may be raised about the safety and well-being of any dependents in the family. Often, this means being prepared to provide referrals or to act as a resource person to find effective and appropriate information.

A fourth question that could be raised, “do I look for ways to keep the family updated on the participant, client or patient in accordance with privacy protections?”(21). While this may be delicate and must be done with full legal compliance, keeping the family updated is an important aim. Depending on the circumstances, these opportunities may help reinforce the family's involvement and inclusion in the life of their loved one or household member.

These questions are important to consider since they help practitioners apply the context of the Checklist into the quality of the programs offered during implementation, and may become part of implementation fidelity testing to assure appropriate perspectives are considered, and can serve basic evaluation needs as well, particularly for process evaluation activities that emerge from the Checklist.

## Limitations

As with all research, we note some limitations of this work. First, our Delphi expert panel included community-based practitioners. We purposively recruited this sample as they were the primary intended Checklist users. While participants were experts in public health practice and implementation, most were not experts in family science. Finding experts in both public health practice and family science is a rare combination. Our goal was to develop a Checklist that would be useful in practice settings regardless of level of expertise in family science. Additionally, experts in family science developed the original Family Impact Checklist and our team of authors had expertise in families and public health. Thus, we prioritized expertise in public health practice for our participants, with the intention that the authors would adjudicate responses to ensure that revisions fit with family science and public health principles.

Relatedly, potential secondary users of the Checklist, including family practitioners and other clinicians, were underrepresented in the Delphi panel. Future research should test this Checklist for use in clinical settings.

Another limitation is that only 50% of our panel responded in Delphi round 2. Historically, Delphi studies often experience low retention rates due to the multiple rounds of participation required (29, 34). Potential reasons for 50% of our participants dropping out after the first round include fatigue, competing demands on their time, and unfamiliarity with the research methods (29). Additionally, some participants may have been uncomfortable posting comments on the Google doc as this phase was not anonymous. Those who did respond to the second round represented a wide range of agencies.

Finally, the Public Health Family Impact Checklist is a revised version of the Family Impact Checklist (27). Although theoretically sound, the reliability of the original checklist and its efficacy in improving family-focused policy and practice are unknown. However, to our knowledge, it was the most relevant and theoretically-based tool for helping practitioners *think family* on a population level. A practical next research step would be to examine the efficacy in practice and the reliability and validity of the revised Checklist tool.

## Conclusion

Families are vital to the health of individuals, communities, and nations. Therefore, it is essential that public health practice target and support families in its programming efforts. However, public health practitioners rarely have training in family science (e.g., family theories, relationships, routines, and other dynamics) and may feel unqualified to develop programming that supports families. The Public Health Family Impact Checklist was developed as a tool for public health professionals to examine the degree to which their program supports families across four principles: family engagement, family responsibility, family stability and family diversity. It is intended for use by public health professionals regardless of their level of training in family issues. By using the Checklist for programs as they are being planned or to make adjustments to existing programs, practitioners will be able to implement programs that better serve families. Ultimately, as practitioners learn to *think family*, this will lead to more sustainable programs and greater strides in improving population health.

## Author Contributions

AC conceived of the study, oversaw the development of the checklist along with the pilot and Delphi data collection process, wrote the sections relating to results, and edited the whole manuscript. LN assisted with the Delphi data collection process, wrote the methods section, and helped edit the entire manuscript. CH helped conceive of the study, assisted in the piloting of the tool, and wrote section Tools to Help Practitioners Involve Families in Public Health Programming. MB helped conceive of the study, helped with the design of the Delphi methodology, and wrote the section on next steps. MN helped conceive of the study and wrote the section on why families matter to public health practice. All authors participated in the interpretation of results and review of the entire manuscript.

### Conflict of Interest

The authors declare that the research was conducted in the absence of any commercial or financial relationships that could be construed as a potential conflict of interest.
