# Supplementary material for: The Public Health Family Impact Checklist: A Tool to Help Practitioners Think Family
Source: Front Public Health. 2019 Nov 12;7:331. doi: 10.3389/fpubh.2019.00331 (PMC6861214; doi:10.3389/fpubh.2019.00331)
Supplement: Supplementary file 1 [file Data_Sheet_1.pdf]

Supplement 1 - Public Health Family Impact Checklist

The purpose of this checklist[1] is to help public health practitioners and their stakeholders to consider how a planned or current program supports and involves families (AKA: "think family"). Families are the primary influencer of many health behaviors and outcomes. Involving the family in public health programs can be complex. Providers can use this checklist to consider how a program can more effectively address a health issue by involving families. This checklist can be used for programs targeting populations of all family types or as the program defines family.

***Instructions**: Answer the background questions first to help you consider how families affect the health issue or problem your program is addressing. After completing the background questions, answer each of the checklist questions by indicating how well you feel that the program follows the "think family" principle. Circle "N/A" if you do not feel that the principle applies to your program.*

**Background Questions**	
What is the name of your program?	
Is the program in the planning stages, or is it currently being implemented?	a. Planning Stage<br>b. Implementation
What health issue(s), health behavior(s) or environmental changes does your program target?	
Based on current research or your experience, what family behaviors and dynamics affect the health issue?	
How does the proposed or current program involve the family or target (either directly or indirectly) family behaviors and dynamics that underlie the health issue?	

[1] This checklist and process has been adapted from Bogenschneider, K., Little, O. M., Ooms, T., Benning, S., Cadigan, K., & Corbett, T. (2012). The family impact lens: A family-focused, evidence-informed approach to policy and practice. *Family Relations*, *61*(3), 514-531.

**Family Engagement** Public health practitioners who "think family" ensure that families are actively involved in all programming phases.				
To what degree are/were families involved in the development and planning of the program?<br>• E.g., Through focus groups, interviews, community meetings, families as volunteers, etc.	Very Well	Some what Well	Not At All	N/A
How well does the program consider and provide services that support the whole family as a unit, including extended family where appropriate?<br>• E.g., Not just mother and child, but a variety of caregivers, siblings, extended family as appropriate.	Very Well	Some what Well	Not At All	N/A
How well does the program connect families to community resources related to the target health issue and help them become informed consumers of these resources?<br>• E.g., Connect families to services with external programs and organizations and track follow-up to assure connection and usage of resources.	Very Well	Some what Well	Not At All	N/A
How well does the program help families build essential social support to address the health issue?<br>• E.g., Social support may include friends, community/ neighborhood, faith-based, etc.	Very Well	Some what Well	Not At All	N/A
How well does the program include families in program evaluation?<br>• E.g., Evaluate changes in family knowledge, attitudes, behaviors, routines, and/or relationships.<br>• E.g., Invite families to help interpret evaluation findings.	Very Well	Some what Well	Not At All	N/A
**Family Responsibility** Public health practitioners who "think family" plan and deliver programs that support and empower family members to perform their responsibilites. The program also supports the family's choices in performing these responsibilities. Examples of such functions may include family formation, partner relationships, economic and financial support, childrearing, and caregiving.				
How well does the program train and encourage staff and partners to support families to make their own decisions and respect family choices?<br>• E.g., Respect family decisions/choices about how much they engage in program activities.	Very Well	Some what Well	Not At All	N/A
How well does the program help families build the capacity to address their health needs without overstepping important boundaries necessary for healthy participant independence?<br>• E.g., Support families without taking over parental roles.	Very Well	Some what Well	Not At All	N/A
How well does the program address participant needs and allow for a balance between work, family, and community commitments?<br>• E.g., Are there requirements that participants must adhere to in order to receive program services that may hinder their employment, such as extensive time waiting in lines or attending trainings during a typical work day?<br>• E.g., Are flexible program options available that make the program accessible to families in various circumstances, such as evening hours for working parents?	Very Well	Some what Well	Not At All	N/A

**<u>Family Stability</u>** Public health practitioners who "think family" strive to plan and deliver programs that encourage stability within the family and recognize the importance of family relationships to individual and family health.				
How well does the program help families prevent health problems before they become serious and affect the family's ability to address the health issue/behavior?<br>• E.g., Education about diabetes treatment, prevention, and resources when a family member is first diagnosed rather than waiting for a diabetic emergency to get added services.<br>• E.g., Include an Adverse Childhood Experience (ACE) screening to help identify if trauma could be a contributor to future health issues.	Very Well	Some what Well	Not At All	N/A
How well does the program help families maintain healthy routines when experiencing stressful conditions or times of change that may be related to the health issue/behavior?<br>• E.g., Help families to maintain family traditions and regular activities when a child is diagnosed with a chronic illness.	Very Well	Some what Well	Not At All	N/A
How well does the program recognize that major changes in family relationships and functionality can impact health, may extend over time, and require major support and attention?<br>• E.g., Divorce, death, marriage, extended family moving in and out of the home, changes in financial status, and other positive and adverse events can cause stress, affect mental health, and change family routines and behaviors. The effects can be short-term or last for years or generations.	Very Well	Some what Well	Not At All	N/A
How well does the program facilitate healthy family relationships and recognize that individual development, wellbeing, and behavior change are profoundly affected by family relationships?<br>• E.g., Parental illness such as depression may affect their caregiving attitudes/behaviors and their ability to maintain healthy family routines.	Very Well	Some what Well	Not At All	N/A
**<u>Family Diversity</u>** Public health practitioners who "think family" understand that programs can have varied effects on families from different cultures and ethnic backgrounds. Through the program, practitioners acknowledge and respect the diversity of families and do not discriminate against or penalize families based on economic situation, educational attainment, family structure, geographic locale, disability, religious affiliation, or gender and sexual minority status of individual family members.				
How well does the program provide services that are available and accessible to diverse families and family types?<br>• E.g., Culturally sensitive, available to families with special needs, geographically reachable, available to working parents outside of normal working hours, affordable, available in the family's native language, etc.	Very Well	Some what Well	Not At All	N/A
How well does the program address root causes of the health issue?<br>• E.g., Social determinants such as social/psychological, economic, political, etc.<br>• E.g., Root causes may include parental involvement, family history of the problem behavior, strength of family bonds, exposure to trauma, other adverse experiences, etc.	Very Well	Some what Well	Not At All	N/A
